# Organophosphate and carbamate susceptibility profiling of Anopheles gambiae sl. across different ecosystems in southern Benin

**DOI:** 10.12688/wellcomeopenres.21452.1

**Published:** 2024-08-05

**Authors:** Camille Dossou, Genevieve Tchigossou, Massioudou Koto, Seun Michael Atoyebi, Eric Tossou, Danahé Adanzounon, Sandra Ateutchia Ngouanet, Haziz Sina, Innocent Djègbè, Adam Gbankoto, Charles Wondji, Rousseau Djouaka

**Affiliations:** 1 Agroecohealth Platform, International Institute of Tropical Agriculture (IITA-Benin), Cotonou, 08 BP 0932 Tri Postal, Benin; 2 University of Abomey-Calavi, Cotonou, BP 526, Benin; 3 National University of Sciences, Technologies, Engineering and Mathematics, Ecole Normale Supérieure de Natitingou, Natitingou, BP 123, Benin; 4 Centre for Research in Infectious Diseases, Yaoundé, Cameroon; 5 Liverpool School of Tropical Medicine, Pembroke Place, Liverpool, L3 5QA, UK

**Keywords:** Anopheles coluzzii, insecticides resistance, bendiocarb, malathion, pirimiphos-methyl, rice field, pineapple field, Benin

## Abstract

**Background:**

To overcome the spread of high pyrethroid resistance in the main malaria vectors and malaria disease persistence, it is crucial to look for effective and better resistance management strategies. Understanding the phenotypic profile of *Anopheles gambiae sl.* against alternatives insecticides like organophosphates and carbamates is crucial.

**Methods:**

*Anopheles* larvae and pupae were collected from the breeding sites in rice fields, pineapple crop areas, and peri-urban areas. WHO susceptibility tests were conducted on unfed female mosquitoes aged 3–5 days old. Mosquitoes were exposed to malathion 5%, pirimiphos-methyl 0.25%, and bendiocarb 0.1% using the standard WHO protocol. Polymerase chain reaction (PCR) techniques were used to detect species, *kdr* and *Ace-1* mutations.

**Results:**

*Anopheles gambiae sl.* from Sèdjè-Dénou rice field population was resistant to bendiocarb (0.1%) with a mortality rate of 72.2% whereas *Anopheles gambiae sl.* populations from Zinvié-Dokomey (rice field), Zè-Tozounmè (pineapple field), and Adjagbo (peri-urban area) were suspected to be resistant with mortality rates of 90%, 93.5%, 95.4% respectively. However, all of them were susceptible to organophosphates (malathion and pirimiphos-methyl) with a mortality rate of 100%. PCR assay revealed that 100% of the mosquitoes tested were *Anopheles coluzzii*. The frequencies of *Ace-1R* mutation in all *Anopheles coluzzii* populations tested were low (3–27%).

**Conclusions:**

Organophosphates (malathion and pirimiphos-methyl) have maintained their efficacy against *Anopheles coluzzii* populations from Sèdjè-Dénou (rice field), Zè Tozounmè (pineapple field), Zinvié Dokomey (rice field), or Adjagbo (peri-urban area). The good efficacy of these organophosphates against *Anopheles coluzzii* populations from the southern part of Benin are observed in the current study. The use of pirimiphos-methyl for IRS in this part of the country would be a successful alternative for malaria control in this area.

## Plain Language Summary

To better manage the spread of high pyrethroid resistance in malaria-carrying mosquitoes, we need to find effective solutions. To do this, we need to understand how these mosquitoes react to different insecticides. We collected *Anopheles* larvae and pupae from different breeding sites and tested them using WHO susceptibility tests. We used malathion, pirimiphos-methyl, and bendiocarb to see how the mosquitoes reacted. We found that *Anopheles gambiae sl*. mosquitoes from different areas had different levels of resistance to bendiocarb. From one of the site, mosquitoes were found resistant, with a mortality rate of 72.2%, while populations from other areas had mortality rates of 90%, 93.5%, and 95.4%, respectively. However, all populations were susceptible to malathion and pirimiphos-methyl. We also used PCR assays to identify the species and mutations present. We found that all of the mosquitoes tested were *Anopheles coluzzii*, and that the mutations present were rare. Our study found that malathion and pirimiphos-methyl are effective against *Anopheles coluzzii* populations in different areas. We suggest using pirimiphos-methyl as a successful alternative for malaria control in this part of the country.

## Background

Malaria persists as a major public health problem in sub-Saharan Africa ^1^. Benin, like most countries in sub-Saharan regions still faces a substantial burden of malaria. Indeed, malaria persists as the primary reason for hospital visits and admissions. While the prevalence of malaria is 15% in the general population, this rate is even higher among children under five years of age (37.2%) ^2^. Effective vector control stands as a crucial aspect in combating this disease. Presently, the primary tools for vector control predominantly consist of long-lasting insecticidal nets (LLINs) and indoor residual spraying (IRS). Unfortunately, malaria vectors have developed resistance to the different classes of insecticides used in vector control. According to the malaria report in 2022, among the 88 countries affected by malaria and reporting data from 2010 to 2020, 78 have identified resistance in at least one malaria-carrying insect to at least one class of insecticide at specific collection sites ^1^. Specifically, 29 countries have noted resistance to pyrethroids, organochlorines, carbamates, and organophosphates across various locations, while 19 have confirmed resistance to all four classes at least once in a local vector at a specific site ^1^. Pyrethroids are currently the only insecticides used to impregnate bed nets. It is clear that, because resistance to these compounds is widespread in Africa ^3– 6^ interest in using IRS (Indoor Residual Spraying) to control malaria vectors is increasing. This IRS strategy is mainly based on using organophosphates and carbamates, either alone or in combination with pyrethroid-impregnated bed nets ^7^. Agriculture has always been designated as the main factors causing insecticide resistance in malaria vector and crop type was among the first factors recognized as modifying the relationship between agricultural insecticide use and insecticide resistance in malaria vectors ^4^.

Some of the earliest reports of insecticide resistance in Africa observed that agricultural insecticide use might have contributed to the selective pressure on anopheline mosquitoes ^8– 11^. Moreover, Nwane *et al*. ^12^ found that mosquitoes from agricultural area with a higher proportion of organophosphate and pyrethroid use had higher levels of resistance to those agents than mosquitoes from this kind of area. In Benin, Yadouléton *et al*. ^13^ and Talom *et al*. ^10^ found vector population resistance levels that correlated directly with reported insecticide use in different pest management strategies. In Benin, the National Malaria Control Program has implemented indoor residual spray using bendiocarb since 2011 and some studies have already reported resistance to this insecticide in malaria vectors ^14^. In addition, *Anopheles gambiae* sensitivity to organophosphates, like fenitrothion, has shown a decline ^14^. Notably resistance to pirimiphos-methyl has not been reported to date, despite its application in Atacora, a northern part of Benin, for IRS between 2013 and 2016 ^15^, however, it' is important to recognize that resistance remains a dynamic issue. There is a continuous risk of its emergence, as highlighted by findings from authors in various other countries ^16, 17^. It is important to manage the evolution of resistance to carbamates and organophosphates in malaria vectors and identify the factors such as crop type driven rapidly by this resistance for sustainable efficacy of malaria control tools. This work aimed to evaluate the insecticide susceptibility profile of malaria vectors from rice- and pineapple-growing environments under high agricultural insecticide pressure and to determine the mechanisms involved.

## Methods

**Figure 1.**
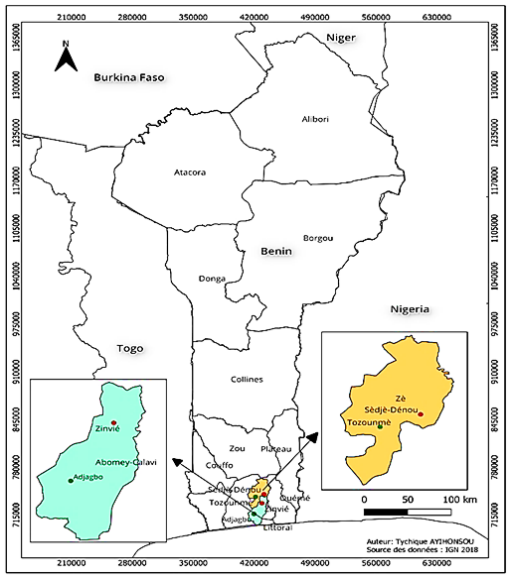
Map of the sampling sites. The study sites are represented by the red and green points. The map was constructed for this publication in QGIS 3.26 ( https://www.norbit.de/~jef/QGIS-OSGeo4W-3.26.3-1.msi?US) using country and regional boundaries from IGN ( https://www.geobenin.bj/carto/www/index.php).

The study was carried out in four localities (Adjagbo, Zè Tozounmè, Zinvié Dokodji and Sèdjè-Dénou) across two communes in southern Benin (Abomey-Calavi and Zè) between June and August 2020 ( Figure 1). These two communes were selected due to the intensive or low agricultural activities conducted by these localities.

Abomey-Calavi is located in the Atlantic department of the Republic of Benin, situated between 6°22’ and 6°30’ north latitude and 2°15’ and 2°22’ east longitude. Geographically, it borders the commune of Zè to the north, the Atlantic Ocean to the south, and is bounded by the communes of Cotonou and So-Ava to the east, while to the west, it shares boundaries with the communes of Ouidah and Tori-Bossito. Its subequatorial climate is characterized by four seasons: a major rainy season (April to July); a small rainy season (September to November); a major dry season (December to March); and a small dry season (August to September). The geomorphology of the Abomey-Calavi municipality reveals a relatively flat relief. The primary features of this relief include: a plain consisting of a sandy strip with both recent and ancient coastal ridges; a plateau of clay soil separated from the plain by the Djonou lagoon and Lake Nokoué; depressions and marshlands in areas situated along the banks of the lake and lagoon. In this commune, the rice production is located at Zinvié with intensive use of agrochemicals meanwhile there is no agrochemical use at Adjagbo which is a peri-urban site ^18^.

The Municipality of Zè is situated in the Atlantic department and is situated between the parallels 6°32’ and 6°87’ north latitude, and between 2°13 and 2°26 east longitude. It is bordered to the north by the Communes of Zogbodomey and Toffo, to the south by the Communes of Abomey-Calavi and Tori-Bossito, to the east by the Communes of Adjohoun and Bonou, and to the west by the Commune of Allada. The climate is subequatorial, marked by more or less high rainfall levels, a relatively low annual thermal amplitude (less than 5°C) and by the succession of four seasons distinct: a long rainy season from April to July; a short rainy season from September to November, a large dry season from December to March and a small dry season in the month of August. The hydrographic network is not dense and is very localized. Only the northern zone of the commune is irrigated by the tributaries of the Ouémé River called Sô. Several shallows dot the territory of the municipality. The main economic activities are agriculture, fishing, product processing agriculture, livestock, commerce, crafts and tourism. In this commune, the rice production is located at Sèdjè-Dénou with intensive use of agrochemicals meanwhile there is less use of agrochemicals at Zè-Tozounmè which is a pineapple production site ^18^.

## Mosquito larvae collection and rearing

*Anopheles* mosquito larvae were collected from June to August 2019 during rice transplanting inside Sèdjè-Dénou rice fields using standard dippers and containers. The collected larvae were sorted, kept in labelled bowls, and transported to the insectary of the International Institute of Tropical Agriculture (IITA) for rearing. Emerging adults from the field-collected larvae, which were placed in cages, were fed on 10% sugar solution and kept at 27 ± 2 °C and relative humidity of 70 ± 10%. Morphologically identified 3 to 5-day-old adult females were used for susceptibility testing to various insecticides and molecular analysis.

## Mosquito genomic DNA extraction and species identification

Genomic DNA of tested mosquitoes was extracted according to the method previously described by Livak ^19^. In brief, whole mosquitoes were ground individually in 100 *μ*L of preheated Livak buffer in 1.5 ml Eppendorf tube and incubated at 65°C for 30 min. A total of 14 *μ*L of K-acetate (8M) were added and the resulting mixture was incubated on ice for 30 min before being centrifuged for 20 min at 12,000 rpm. The supernatant was pipetted into a new 1.5 mL Eppendorf tube to which 200 *μ*L of ethanol (100%) was added; the mixture was centrifuged for 15 min at 12,000 rpm to precipitate the DNA. The supernatant was discarded subsequently and the DNA pellet formed at the bottom of tubes was purified with 100 µL ice-cold ethanol (70%). The DNA pellet was dried on the bench for 1 hour. The extracted DNA was reconstituted in 50 *μ*L DNase-free water (Sigma-Aldrich, United Kingdom). DNA concentration and purity of the samples were read using the nanodrop (Thermo scientific, CA, USA). prior to storage at -20°C.

## Identification of *Anopheles gambiae sl* subspecies

The different species of *Anopheles gambiae sl.* ( *Anopheles gambiae ss* and *Anopheles coluzzii*) were determined using SINE-PCR ^20^. The following primers were used: Forward primer 5’-TCGCCTTAGACCTTGCGTTA-3’ and the reverse primer 5’-CGCTTCAAGAATTCGAGATAC-3’. The PCR took place in a thermocycler (Gradient Thermal cycler; Gene Pro Scientific Instruments Co., Ltd Hangzhou, P.R. China) according to the following programme: 94°C for 5min, 94°C for 25 s, and 54°C for 30 s; 72°C for 1 min repeated 35 times; and a final step at 72°C for 10 min to terminate the reaction. The agarose gel was prepared at 1.5% in TAE (Tris/acetate/EDTA) containing Midori green. The PCR product was loaded on gel and allowed to migrate under a voltage of 100 V for 45 min. The result was visualized with a UV illuminator (Thermo scientific, CA, USA). Expected bands by species was 479 bp for *Anopheles gambiae coluzzii* and 249 bp for *Anopheles gambiae*.

## Insecticide bioassay and mosquito conservation

The susceptibility of mosquitoes was assessed through the WHO cylinder test and the mosquitoes used for the test were *Anopheles gambiae s.l* wild strain species. The susceptibility test performed according to the WHO protocol ^21^ involved exposure of 3–5-days old nonblood-fed female adults to a diagnostic dosage of the following insecticides: bendiocarb (0.1%), malathion (5%), and pirimiphos-methyl (0.25 %). *Anopheles gambiae Kisumu* strain was used as the reference susceptible strain and was tested simultaneously with field mosquitoes. For each insecticide test, at least 100 mosquitoes were used.

## Identification of resistance genes

The real-time PCR was used to investigate the presence of insecticide resistance genes including *kdr-East*, *West*, *N1575Y* and *Ace-1* ^22^. The reaction was carried out in an Agilent Stratagene MX3000 qPCR thermocycler (Agilent Technologies, Santa Clara, CA, USA). For kdr genotyping, Forward and Reverse primers [Forward (5'-CATTTTTCTTGGCCACTGTAGTGAT-3'), and Reverse (5'- CGATCTTGGTCCATGTTAATTTGCA-3')] and minor groove binding (MGB) probes (Applied Biosystems) were used. The probe WT (5'- CTTACGACTAAATTTC-3') was labelled with HEX at the 5' end for the detection of the wild type allele, while the probe kdr-W (5'- ACGACAAAATTTC-3') were labelled with FAM for detection of the kdr-L1014F allele. For G119S genotyping, a universal primer G119-Reverse (5'- CGGTGGTCGTACACGTCCAGGGT-3') that anneals to both resistant and susceptible allele as well as the primer G119S-Forward (5'-GCGGGCAGGGCGGCGGGGGCGGGGCCCTGTGGATCTTCGGCGGCG-3') that specifically anneals to the susceptible allele and primer G119R-Forward (5'-GCGGGCCTGTGGATCTTCGGCGGCA-3') that specifically anneals to the resistant allele were used. Briefly, each reaction was conducted in a total volume of 10 µl that comprise of 5 µl Sensimix (Meridian BioScience), 0.125 µl of 40x Probe Mix coupled to allelic-specific primers, 3.875 µl of dH20, and 1 µl of genomic DNA. Thermocycling conditions were set at an initial 95°C for 10 min, followed by 40 cycles each of 95°C for 10 sec, and 60°C for 45 sec.. Genotypes were scored from dual color scatter plots produced by the device after reaction.

## Data analysis

The mortality of the different tests achieved was interpreted according to the criteria proposed by 21 as follows: mortality between 98% and 100% indicates that the vectors are susceptible, mortality between 90% and 97% indicates the suspected resistance in the vector population which must be confirmed, and mortality less than 90% indicates that mosquito population is resistant.

The resistance ratio (RR) of vectors to the various insecticides was determined from reports of the knock-down time of 50% of the population (KDT50) of wild mosquitoes and those of the susceptible *Anopheles gambiae Kisumu* strain and the knock-down time of 95% of the population (KDT95) of wild mosquitoes and those of the susceptible *Anopheles gambiae Kisumu* strain. This ratio expresses the level of resistance of the field strain compared with the susceptible *Anopheles gambiae Kisumu* strain based on the knock-down effect. The time at which 50% of the test population were knocked down (KDT50) or 95% of the test population were knocked down (KDT95) was determined using R software ^23^, via log-probit analysis, with a level of a significant set at a p value less than 0.05. Values of resistance ratio (RR) greater than 5 are an indication of resistance and values less than or equal to 5 are considered as susceptible ^21^.

## Results

### Molecular species

Overall, 800 *Anopheles gambiae sl.* specimens were characterized. In the all-study sites, all mosquito specimens analysed were *Anopheles coluzzii*.

### Resistance ratio and knock down

The 50% knockdown times (KDT50) were determined against three insecticides. In all study sites (except Adjagbo), the fastest knockdown time (KDT50) was recorded in malathion followed by pirimiphos-methyl and bendiocarb. The fastest knockdown mosquito (KDT50 = 20.615) was recorded in Zè-Tozounmè with malathion, whereas the slowest (KDT50 = 42.329) was recorded in Sèdè-Dénou with Bendiocarb ( Table 1).

**Table 1.** Resistance ratio (RR50) of mosquito populations ( *Anopheles gambiae*) to bendiocarb, malathion, and pirimiphos-methyl. CI50: confidence interval at 50%; KdT50: knock down time of 50% of the population; *KdT50* of the wild strain divided by KdT50 of the Kisumu reference strain; RR50: resistance ratio at 50%.

Insecticides by locality	Kdt _50_ (CI _50_) Kisimu (min)	Kdt _50_ (CI _50_) wild strain(min)	RR _50_
*Adjagbo*			
Bendiocarb (0.01 %)	10.955 (10.206 - 11.695)	28.965 (26.766 - 31.049)	2,644
Malathion (5 %)	11.715 (10.924 - 12.503)	23.688 (20.125 - 26.922)	2,022
Pirimiphos-methyl (0.25 %)	8.969 (8.009 - 9.863)	21.940 (20.276 - 24.454)	2,446
*Zinvié Dokomey*			
Bendiocarb (0.01 %)	10.955 (10.206 - 11.695)	31.977 (30.785- 33.134)	2,919
Malathion (5 %)	11.715 (10.924 - 12.503)	22.255 (19.810 - 24.577)	1,899
Pirimiphos-methyl (0.25 %)	8.969 (8.009 - 9.863)	25.933 (24.010 - 27.802)	2,891
*Zè Tozounmè*			
Bendiocarb (0.01 %)	10.955 (10.206 - 11.695)	28.793 (27.095 - 30.444)	2,628
Malathion (5 %)	11.715 (10.924 - 12.503)	20.615 (18.378 - 22.725)	1,760
Pirimiphos-methyl (0.25 %)	8.969 (8.009 - 9.863)	22.251 (19.610 - 25.017)	2,481
*Sèdjè-Dénou*			
Bendiocarb (0.01 %)	10.955 (10.206 - 11.695)	42.329 (39.822 - 45.205)	3.864
Malathion (5 %)	11.715 (10.924 - 12.503)	31.692 (20.260 - 48.610)	2.705
Pirimiphos-methyl (0.25 %)	8.969 (8.009 - 9.863)	34.863 (33.725 - 35.973)	3.887

Concerning the 95% knockdown times (KDT95) in all study sites, the fastest knockdown time (KDT95) was recorded with malathion followed by pirimiphos-methyl and bendiocarb. The fastest knockdown mosquito (KDT95 = 36.692) was recorded in Zè Tozounmè with malathion, whereas the slowest (KDT95 = 106.133) was recorded in Sèdè-Dénou with Bendiocarb as for KDT50 ( Table 2). The RR95 varied among localities. The RR95 of bendiocarb varied between 3.093 and 6.606. The highest RR95 of bendiocarb was recorded at Sèdjè-Dénou. Concerning malathion, the RR95 varied between 2.138 and 2.825 with 100% of the mosquitoes of the four localities being knocked down before the end of the exposure time. The same trends were observed with pirimiphos-methyl with 100% of the mosquitoes of the four localities being knocked down at the end of the exposure time. The RR95 varied between 2.738 and 3.737.

**Table 2.** Resistance ratio (RR95) of mosquito populations ( *Anopheles gambiae*) to bendiocarb, malathion, and pirimiphos-methyl. CI95: confidence interval at 95%; KdT95: knock down time of 95% of the population; *KdT95* of the wild strain divided by KdT95 of the Kisumu reference strain; RR95: resistance ratio at 95%.

Insecticides by locality	Kdt _95_ (CI _95_) Kisumu (min)	Kdt _95_ (CI _95_) wild strain(min)	RR _95_
*Adjagbo*			
Bendiocarb (0.01 %)	16.066 (14.574 - 18.858)	49.692 (43.849 - 60.824)	3.093
Malathion (5 %)	17.158 (15.844 - 19.236)	44.547 (38.389 - 56.882)	2.596
Pirimiphos-methyl (0.25 %)	16.981 (15.032 - 20.167)	46.488 (41.915 - 54.542)	2.738
*Zinvié Dokomey*			
Bendiocarb (0.01 %)	16.066 (14.574 - 18.858)	58.373 (49.675 - 73.635)	3.633
Malathion (5 %)	17.158 (15.844 - 19.236)	45.937 (43.428 - 49.470)	2.677
Pirimiphos-methyl (0.25 %)	16.981 (15.032 - 20.167)	56.399 (51.084 - 64.692)	3.321
*Zè Tozounmè*			
Bendiocarb (0.01 %)	16.066 (14.574 - 18.858)	51.383 (46.775 - 58.467)	3.192
Malathion (5 %)	17.158 (15.844 - 19.236)	36.692 (33.060 - 42.482)	2.138
Pirimiphos-methyl (0.25 %)	16.981 (15.032 - 20.167)	48.448 (41.636 - 60.437)	2.853
*Sèdjè-Dénou*			
Bendiocarb (0.01 %)	16.066 (14.574 - 18.858)	106.133 (90.373 - 133.126)	6.606
Malathion (5 %)	17.158 (15.844 - 19.236)	48.475 (46.091 - 51.794)	2.825
Pirimiphos-methyl (0.25 %)	16.981 (15.032 - 20.167)	63.459 (50.639 - 95.960)	3.737

## Susceptibility of *Anopheles gambiae sl.* to carbamates and organophosphates

**Figure 2.**
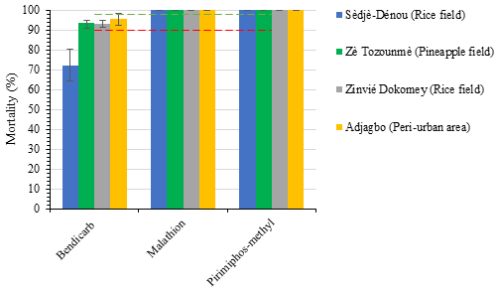
Mean percentage mortalities of *Anopheles gambiae s.l*. mosquitoes from the four study sites after 24 hours post exposure period. Data are shown as mean ± standard error of the mean (SEM). Red corresponds to 90% mortality and green line to 98% mortality.

The result of the susceptibility assays revealed that the percentage mortalities of *Anopheles* mosquito exposed to organophosphate insecticides (Malathion and Pirimiphos methyl) were higher than those of carbamate (Bendiocarb) insecticides ( Figure 2). After 24 hours post exposure period, *Anopheles* populations from all study sites were fully susceptible (100% mortality) to Malathion and Pirimiphos methyl. The lowest mortality rates (Sèdjè-Dénou 72.2%; Zè Tozounmè 93.4%; Zinvié Dokomey 93.0%; Adjagbo 95.4%) were obtained from exposures of mosquito populations to bendiocarb (carbamate) insecticide ( Figure 2).

## Resistance genes

**Figure 3.**
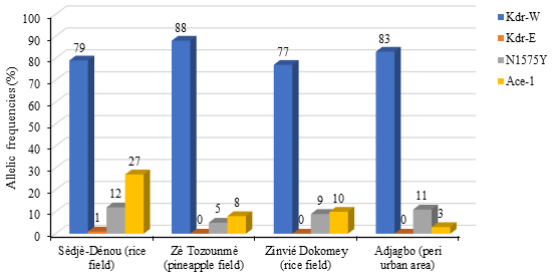
Allelic frequencies of Kdr-W, Kdr-E, N1575Y and Ace-1 in *Anopheles coluzzii* populations.

The *Kdr-East*, *Kdr-West*, *N1575Y* and *Ace- 1* genes mutation were genotyped. Among these resistance genes mutation, *kdr-West* was the most frequently expressed within the population of vectors with an allelic frequency ranged between 77% (Sèdjè-Dénou) and 88% (Zè Tozounmè). *Kdr-East* mutated genotype was expressed in any *Anopheles coluzzii* specimen analysed apart from Sèdjè strain (1%). *N1575Y* mutated genotype was faintly expressed in the *Anopheles coluzzii* specimen analysed. The allelic frequency ranged between 5% and 12% with the highest frequency recorded Sèdjè-Dénou *Anopheles coluzzii* mosquitoes population. Concerning the *Ace-1* mutation, the allelic frequency of the mutant gene ranged between 8% and 27% with the highest frequency recorded Sèdjè-Dénou *Anopheles coluzzii* mosquitoes population ( Figure 3).

## Discussion

This study assessed the susceptibility status of *Anopheles* mosquito populations to organophosphate and carbamate insecticides across variable agroecosystems in southern Benin. Similarly, to other reports, *Anopheles coluzzii* was detected as the main vector for malaria in the investigated study sites ^24– 26^. Findings showed that this *Anopheles coluzzii* population is developing an insensitivity to carbamate (bendiocarb) but remains fully susceptible to organophosphates (malathion and pirimiphos-methyl). In Benin, multiple resistance to insecticides mainly pyrethroids and carbamates have been reported ^24– 29^ as observed in this study. This raises serious concerns about the future use of long-lasting insecticidal nets (LLIN) and indoor residual spraying (IRS) for malaria vector control. In this context, the research of an alternative approach based on entomological survey is important.

In the present study, the KDT50 and KDT95 values obtained with *Anopheles gambiae Kisumu* susceptible reference strain was lower than those recorded with the wild populations of *Anopheles coluzzii* from Adjagbo (peri-urban area). Therefore, *Anopheles coluzzii* from Adjagbo took more time to die when they were exposed to carbamate (bendiocarb) compared to *Anopheles gambiae Kisumu* susceptible strain. The wild population in Adjagbo were also found to have high levels of organophosphate (pyrimiphos-methyl and malathion). Nevertheless, the RR50 or RR95 didn’t clearly indicate insecticides resistance in this *Anopheles coluzzii* population. On the other hand, the same trends were almost observed with *Anopheles coluzzii* populations from the rice and pineapple crop production except for bendiocarb susceptibility among Sèdjè-Dénou *Anopheles coluzzii* population. The KDT95 values obtained with the wild populations of *Anopheles coluzzii* from Sèdjè-Dénou rice field was 6 times higher than those recorded with *Anopheles gambiae Kisumu* susceptible strain. This clearly indicates bendiocarb resistance in *Anopheles Coluzzii* from Sèdjè-Dénou. This result was confirmed by the mortalities recorded 24 hours after the exposure period.

The bioassay results confirmed resistance to bendiocarb in *Anopheles Coluzzii* from Sèdjè-Dénou but a possible resistance to bendiocarb of each of Adjagbo, Zinvié, and Zè *Anopheles coluzzii* populations. By contrast, full susceptibility to pirimiphos-methyl and malathion was observed in all four study sites. Both carbamate and organophosphate insecticides act on the synapse by inhibiting the action of acetylcholinesterase enzyme ^30^. Specifically, in *Anopheles gambiae sl.* mosquitoes, the acetylcholine-1 *R* (ace-1 *R*) gene had been found to cause cross-resistance between organophosphate and carbamate insecticides ^31^. Therefore, the results of carbamate resistance and full organophosphate insecticide susceptibility observed in this study could raise questions since the general mode of action of carbamate and organophosphate insecticide classes are similar. Nevertheless, a possible reason for the differential *Anopheles gambiae s.l* susceptibility to carbamate and organophosphate observed in this study could be due to the type of enzymes elevated in the mosquitoes through metabolic resistance. It was reported that in rice fields and pineapple fields, herbicides and inorganic fertilizers are largely used than insecticides ^9, 32^. But the use of diverse xenobiotics such as herbicides and fertilizers in rice or pineapple production may impact the metabolic system of mosquito larvae, leading to a wide array of insensitivity to multiple insecticides and favouring the development of resistance across successive generations. Heavy metals have been shown to have an inducing role on enzymes responsible for insecticide degradation in mosquitoes. It was highlighted that selecting *Anopheles gambiae sl.* larvae with a mixture of agrochemicals increased their resistance to a broad range of insecticides at the adult stage ^8, 10, 33– 35^.

According to WHO (2012), though the ace-1 *R* gene has the same high likelihood of modulating for resistance to both carbamates and organophosphates, the presence of elevated monooxygenases is more important in conferring resistance to carbamates than esterases while the presence of elevated esterases are more important in conferring resistance to organophosphates than monooxygenases. Therefore, the possibility of the presence of elevated monooxygenases rather than elevated esterases could be a possible reason responsible for the carbamate resistance and organophosphate susceptibility observed in the *Anopheles gambiae s.l* mosquito populations tested in this study. Further biochemical studies on the enzymes elevated in the resistant *Anopheles* mosquito populations from these study sites are required to confirm this possibility. Similar results of carbamate resistance and full susceptibility of *Anopheles gambiae sl.* to organophosphate observed in this study have been reported by Aïkpon *et al*., ^14^; Kpanou *et al.*, ^36^, Sagbohan *et al.,* ^24^, Zoungbédji *et al*., ^25^, Bouraima *et al*., ^26^ in Benin, in Togo ^37^, Burkina Faso ^38^ and Nigeria ^39^. This susceptibility to organophosphates could also be explained by the low frequency of *Ace-1* resistant allele among the investigated *Anopheles coluzzii* populations.

In the current study, all *Anopheles coluzzii* specimens issued from WHO bioassays, were either homozygous susceptible or heterozygous individuals for *Ace-1* mutation. There were no homozygous resistant individuals. These results might be related to the high fitness cost of the *Ace-1R* mutation, resulting in the death of the homozygous resistant mosquitoes ^40– 42^. In *Anopheles gambiae s.l* populations, the *Ace-1* mutation has been associated with a high fitness cost as the frequency of the *Ace-1* mutation in mosquito populations declines rapidly after a few generations in the absence of selection pressure from organophosphates or carbamates insecticides ^31^.

Apart from Ace-1 mutation, the kdrWest allele (1014F) was found in very high frequencies across the different studied populations, whereas kdr East mutation (1014S) and N1575Y mutation was found in very low frequencies. These findings were in accordance with previous reports suggesting the high distribution of the L1014F allele across the country ^24, 25, 36, 43^.

## Conclusion

Organophosphates (malathion and pirimiphos-methyl) have maintained their efficacy against *Anopheles coluzzii* populations from Sèdjè-Dénou (rice field), Zè Tozounmè (pineapple field), Zinvié Dokomey (rice field) or Adjagbo (peri-urban area). The good efficacy of these organophosphates against *Anopheles coluzzii* populations from the southern part of Benin is clearly observed in the current study. The use of pirimiphos-methyl for IRS in this part of the country would be a successful alternative for malaria control in this area.

## Ethics and consent

Ethical approval and consent were not required.

## Data Availability

Open Science Framework: Organophosphate and carbamate susceptibility profiling of Anopheles gambiae sl. across different ecosystems in southern Benin. https://doi.org/10.17605/OSF.IO/RKTCX ^43^. Data are available under the terms of the Creative Commons Zero "No rights reserved" data waiver (CC0 1.0 Public domain dedication).
