# Supplementary material for: Organophosphate and carbamate susceptibility profiling of Anopheles gambiae sl. across different ecosystems in southern Benin
Source: Wellcome Open Res. 2024 Nov 14;9:424. Originally published 2024 Aug 5. [Version 2] doi: 10.12688/wellcomeopenres.21452.2 (PMC11599805; doi:10.12688/wellcomeopenres.21452.2)
Supplement: Peer review history

# Reviewer report: Reviewer response for version 2

**DOI:** 10.21956/wellcomeopenres.25812.r111653

**Published:** 2024-11-26

**Authors:** Maria Kaiser

**Affiliations:** 1 National Institute for Communicable Diseases., Johannesburg, South Africa

The authors have addressed many of the comments and the figures and tables have been improved. In the plain language summary it is not entirely correct to state that Ace-1 mutations were low as 27% is relatively high. Specifically, it would be prudent to mention that where the Ace-1 frequency was higher there was also a greater proportion of resistant (to bendiocarb) mosquitoes, although the authors have also discussed and made valid points about the possible role of metabolic resistance.

Is the work clearly and accurately presented and does it cite the current literature?

Partly

If applicable, is the statistical analysis and its interpretation appropriate?

Partly

Are all the source data underlying the results available to ensure full reproducibility?

No source data required

Is the study design appropriate and is the work technically sound?

Partly

Are the conclusions drawn adequately supported by the results?

Partly

Are sufficient details of methods and analysis provided to allow replication by others?

No

Reviewer Expertise:

malaria vector control, malaria vector surveillance, vector behaviour, Anopheles

I confirm that I have read this submission and believe that I have an appropriate level of expertise to confirm that it is of an acceptable scientific standard.

# Reviewer report: Reviewer response for version 2

**DOI:** 10.21956/wellcomeopenres.25812.r111652

**Published:** 2024-11-22

**Authors:** Jackline Martin

**Affiliations:** 1 Kilimanjaro Christian Medical University College, Moshi, Tanzania

Approved.

Is the work clearly and accurately presented and does it cite the current literature?

Yes

If applicable, is the statistical analysis and its interpretation appropriate?

Partly

Are all the source data underlying the results available to ensure full reproducibility?

Yes

Is the study design appropriate and is the work technically sound?

Yes

Are the conclusions drawn adequately supported by the results?

Yes

Are sufficient details of methods and analysis provided to allow replication by others?

Yes

Reviewer Expertise:

Entomologist with 12 years experience in evaluation of vector control products in Africa. experienced in malaria vector behavior and host vector interactions

I confirm that I have read this submission and believe that I have an appropriate level of expertise to confirm that it is of an acceptable scientific standard.

# Reviewer report: Reviewer response for version 1

**DOI:** 10.21956/wellcomeopenres.23720.r103873

**Published:** 2024-11-05

**Authors:** Jackline Martin

**Affiliations:** 1 Kilimanjaro Christian Medical University College, Moshi, Tanzania

The study is well-executed, addressing an important gap in malaria vector control in Benin by highlighting the potential of organophosphates like malathion and pirimiphos-methyl in IRS programs. These findings support the strategic rotation or integration of these insecticides to manage resistance and enhance malaria control efficacy.

 Please ensure minor corrections, including the correct notation for An. gambiae s.l. and s.s., where only the species name (An. gambiae) should be italicized, and the abbreviations "s.l." and "s.s." should remain in regular font to maintain consistence.

 Explain how mosquitoes for molecular analysis were stored/preserved.

Is the work clearly and accurately presented and does it cite the current literature?

Yes

If applicable, is the statistical analysis and its interpretation appropriate?

Partly

Are all the source data underlying the results available to ensure full reproducibility?

Yes

Is the study design appropriate and is the work technically sound?

Yes

Are the conclusions drawn adequately supported by the results?

Yes

Are sufficient details of methods and analysis provided to allow replication by others?

Yes

Reviewer Expertise:

Entomologist with 12 years experience in evaluation of vector control products in Africa. experienced in malaria vector behavior and host vector interactions

I confirm that I have read this submission and believe that I have an appropriate level of expertise to confirm that it is of an acceptable scientific standard, however I have significant reservations, as outlined above.

# Response

**Published:** 2024-11-10

**Authors:** Camille DOSSOU

**Affiliations:** International Institute for Tropical Agriculture Benin, Cotonou, Littoral Department, Benin

1) We agree with the Reviewer’s comments about ‘’the correct notation for An. gambaie sl. And ss.’’. We have considered this in the revised manuscript.

 2) The reviewer asked us to explain how mosquitoes for molecular analysis were stored/preserved. More details have been given in the revised manuscript to explain what. Please, refer to the Methods section in the revised manuscript.

# Reviewer report: Reviewer response for version 1

**DOI:** 10.21956/wellcomeopenres.23720.r95964

**Published:** 2024-09-30

**Authors:** Maria Kaiser

**Affiliations:** 1 National Institute for Communicable Diseases., Johannesburg, South Africa

This article looks at populations of Anopheles gambiae s.l. from areas of differing agrochemical use in southern Benin (rice growing areas, pineapple growing area, peri-urban area).  Bednets, impregnated with pyrethroids, have been in use and mosquitoes in the area are resistant to pyrethroids. Therefore, it is important to determine the susceptibility status of the the vector population/s to other insecticide classes which could be used for indoor residual spraying. 

 Anopheline larvae were collected from two districts in southern Benin and within those districts from an area with greater agrochemical use and one from an area with less agrochemical use. Standard WHO tube bioassays against 1 carbamate (bendiocarb 0.1%) insecticide and 2 organophosphate (malathion 5% and pirimiphos methyl 0.25%) insecticides at diagnostic concentrations were conducted. Knockdown 50 and 95% times were also determined and compared to a susceptible reference strain to determine the resistance ratio of each population to the different insecticides. The frequencies of insecticide resistance associated mutations: kdr (east and west); N1575Y, and Ace-1R, were determined. 

 Mosquitoes from all areas tested were found to be susceptible to organophosphates however possible resistance or resistance to the carbamate, bendiocarb was detected and was highest in mosquitoes collected from one of the rice growing areas.

 Kdr-w was detected at high frequencies, kdr-e was detected at a frequency of only 1% in specimens from Sedje-Denou, N1575Y was detected at low frequency and Ace-1R was detected as well and at a higher frequency in the mosquitoes particularly from one of the rice growing areas than in mosquitoes from the other sites. 

**The information presented is valuable. Consider the following for improvements:**

**In plain language summary-**

 It is stated that mutations present were rare- this was not true for all mutations and can be written better to more accurately reflect the frequency of mutations

**In the methods-**

- In describing the study sires communes and municipalities seem to be used interchangeably which can get confusing.
- The quality of Figure 1 should be improved as it is pixelated and difficult to read
- The reason for using red and green dots to portray the different sites is not coming across. From reading about the study sites it appears that the green sites are located where there is likely to be less agrochemical use than in those sites portrayed as red dots. Please indicate dot colour significance in a key or in the figure legend
- during larval rearing to adulthood what food was given to the larvae to complete development (include this). if not fed and they survived off what was already in the water they were collected in then state that
- Include determination of KD50 and 95 in the methods as well as information of the calculations of the respective resistance ratios- it is referred to in data analysis but can go under (or before/after) bioassay section
- It is not clear how morphological identification was performed- include the method and the morphological key/s used (and reference the key/s). 
- The methods presented for the detection of resistance associated mutations were not sufficient for all the mutations which were screened for. Include appropriate references for the detection of each mutation.
- For data analysis section - is there a statistical difference in the frequency of resistance associated mutations between the different sites- this would be valuable to include especially for the Ace-1R mutation
- Include information on controls for the various tests/ assays

**In the results:**

 it is stated that 800 Anopheles gambiae s.l. specimens were molecularly characterised- how many successfully amplified? Include whether any specimens did not amplify as well. This can be given as a percentage of the total screened for species identification

 Include sample sizes for each test/ analysis performed, in all figures and graphs or alternatively clearly stated in the methods

 For the resistance mutations it is not clear if mosquitoes were assayed for the mutations after exposure to insecticides and therefore would be possible to determine any association between resistance mutation (genotype) and survival or death following exposure (phenotype) or if it was a sample not exposed to insecticides which were screened for mutations. Please indicate

 In tables 1 and 2 highlight which RR show resistance by using different font for those RR comparison/s

 Improve the quality of figure 2. It is pixelated. Include the results from unexposed control mosquitoes in the graph or in text

 How many mosquitoes were used from each site to determine mutation/ allelic frequency? include in graph

**Discussion and conclusions-** 

 It is mentioned in the discussion that multiple insecticide resistance was observed in this study- this is not true as only resistance to bendiocarb was detected

 A suggestion is to emphasize the requirement for susceptibility testing of the vector population targeted for IRS in order to determine or select the most appropriate insecticide option for use in a vector control/ IRS programme instead of highlighting organophosphates as alternative insecticide class for IRS 

 Elevated mono-oxygenases vs esterases is discussed consider this in terms of pyrethroid resistance in the area/ population as well

 Include discussion on the higher frequency of Ace-1R detected in samples from one of the rice growing areas. Were differences between the 2 rice growing areas observed that could possibly account for the higher frequency in one rice area than the other (overall rice growing areas had higher Ace-1R frequencies)

Is the work clearly and accurately presented and does it cite the current literature?

Partly

If applicable, is the statistical analysis and its interpretation appropriate?

Partly

Are all the source data underlying the results available to ensure full reproducibility?

No source data required

Is the study design appropriate and is the work technically sound?

Partly

Are the conclusions drawn adequately supported by the results?

Partly

Are sufficient details of methods and analysis provided to allow replication by others?

No

Reviewer Expertise:

malaria vector control, malaria vector surveillance, vector behaviour, Anopheles

I confirm that I have read this submission and believe that I have an appropriate level of expertise to confirm that it is of an acceptable scientific standard, however I have significant reservations, as outlined above.

# Response

**Published:** 2024-11-10

**Authors:** Camille DOSSOU

**Affiliations:** International Institute for Tropical Agriculture Benin, Cotonou, Littoral Department, Benin

**In plain language summary** A sentence was reformulated.

 From “We found that all of the mosquitoes ...... were low”.

**In the methods**

 “Commune” was replaced by “Municipality” throughout the revised manuscript.

 Figure 1

 The quality of Figure 1 has been improved.

 Dot color significance was indicated in the Figure 1 legend the revised manuscript.

 More details have been given to explain how anopheline was fed in the revised manuscript. 

 The morphological identification performed is now indicated including the references of the identification keys used in the revised manuscript.

 The additional references were included about resistance gene genotyping in the revised manuscript. The ‘’data analysis’’ section was reformulated to explain how allelic frequency was calculated and compared between the different surveyed localities in the revised manuscript.

**In the results**

  The ‘’molecular species’’ paragraph was reformulated to give more precisions. The ‘’resistance genes’’ paragraph was reformulated to give more details.
